# Metabolic Effects of Fluoxetine in Adults with Type 2 Diabetes Mellitus: A Meta-Analysis of Randomized Placebo-Controlled Trials

**DOI:** 10.1371/journal.pone.0021551

**Published:** 2011-07-28

**Authors:** Zi Ye, Lili Chen, Zhen Yang, Qin Li, Ying Huang, Min He, Shuo Zhang, Zhaoyun Zhang, Xuanchun Wang, Weiwei Zhao, Ji Hu, Chao Liu, Shen Qu, Renming Hu

**Affiliations:** 1 Institute of Endocrinology and Diabetology, Huashan Hospital, Shanghai Medical College, Fudan University, Shanghai, China; 2 Department of Endocrinology, Shanghai Tenth People's Hospital, School of Medicine, Tongji University, Shanghai, China; 3 Department of Endocrinology, The Second Affiliated Hospital of Soochow University, Suzhou, China; 4 Department of Endocrinology, Jiangsu Province Hospital on Integration of Chinese and Western Medicine, Nanjing University of Chinese Medicine, Nanjing, China; Universita Magna-Graecia di Catanzaro, Italy

## Abstract

**Background:**

The prevalence of obesity and diabetes is increasing dramatically throughout the world. Studies have shown that excess adiposity is a critical predictor of new onset T2DM. This meta-analysis is aimed to assess the metabolic effects of fluoxetine in T2DM.

**Methods and Findings:**

Electronic search was conducted in the database Medline, PubMed, EMBASE, and the Cochrane library, from inception through to March 2011. A systematic review of the studies on the metabolic effects of fluoxetine in T2DM was performed. The weighted mean difference (*WMD*) and its 95% *CI* were calculated from the raw data extracted from the original literature. The software Review Manager (version 4.3.1) and Stata (version 11.0) were applied for meta-analysis. Five randomized, placebo-controlled trials were included in the meta-analysis. According to *WMD* calculation, fluoxetine therapy led to 4.27 Kg of weight loss (95%CI 2.58–5.97, *P*<0.000 01), 1.41 mmol/L of fasting plasma glucose (FPG) decrement (95%CI 0.19–2.64, *P* = 0.02) and 0.54 mmol/L of triglyceride (TG) reduction (95%CI 0.35–0.73, *P*<0.000 01) compared with placebo. Moreover, fluoxetine therapy produced 0.78% of HbA1c decrement (95%CI −0.23–1.78). However, this effect was not statistically significant (*P* = 0.13).

**Conclusions:**

Short period of fluoxetine therapy can lead to weight loss as well as reduction of FPG, HbA1c and TG in T2DM.

## Introduction

The prevalence of obesity and diabetes is increasing dramatically throughout the world [1]. The global prevalence of diabetes in 2010 was 284 million people worldwide constituting around 6.4% of the world population, which is higher than was projected in earlier studies [2]. Furthermore, the projections for 2030 show the prevalence to reach 439 million individuals comprising ∼7.7% of the world population [2]. The burden of diabetes on the world economy has been rising steadily in the last decade to reach $376 billion in 2010 and is expected to reach $490 billion in 2030 [3]. From an epidemiologic point of view, the first relevant point is that almost 80% of diabetes cases could be prevented just by avoiding overweight and obesity [2]. The estimated attributable risk of excess body weight is extremely high; no other modifiable effect has such an impact on the health of the general population.

Studies have shown that excess adiposity is a critical predictor of new onset type 2 diabetes [1], [4], [5], [6]. It has been estimated that every 1 kg increase in weight is associated with a relative 9% increase in diabetes prevalence [7]. T2DM individuals with 20%–30% above their ideal body weight have 2.5–3.0 fold higher mortality than diabetic people at their ideal weight. Concerning the patients of T2DM who are more than 40% above their ideal weight, the mortality increases greater than five fold [8]. Adipose, now recognized as an endocrine and secretory tissue, can release a wide range of inflammation-related adipokines, which are considered to play an important role in the development of T2DM and metabolic syndrome [9], [10].

Weight loss appears to be a more dominant predictor of reduced diabetes incidence compared with changes in diet or activity level [11], [12], [13]. The reduction of fat mass due to weight loss can produce numerous benefits in T2DM, including improved glucose metabolism and increased insulin sensitivity [6], [14], [15], [16]. These benefits are clinically meaningful only when the weight loss is sustained over time. Based on the preceding observations, weight control in type 2 diabetes has been recommended as an early and important intervention [17], [18], [19].

Type 2 diabetic patients under lifestyle intervention will lose, on average, 8% of initial body weight over 3–12 months [20]. However, most obese people regain their weight they have successfully lost from dietary and behavioral treatment in long-term outcome [21], [22]. It seems difficult to achieve sustained weight control in general population [20], [23]. For Type 2 diabetic patients, this issue is more complicated. Studies demonstrated that diabetic patients lost less weight and regained their weight more rapidly compared with the non-diabetic [24], which can be attributed to the physiologic derangement and insulin treatment of T2DM [25]. Hence, in the obese individuals for whom behavioral therapy has failed, medical interventions such as weight-management drugs are required in addition to the usual anti-diabetic treatment. In general, pharmacologic therapy of obesity consists of centrally acting appetite suppressants, drugs with a peripheral effect on appetite, drugs that affect nutrient partitioning, and drugs that increase thermogenesis. Several weight-loss medications have been proved to be effective, and some have been applied in clinical practice. However, only two drugs, orlistat and sibutramine, have been approved by the US Food and Drug Administration (FDA) to treat obesity long term, and both medications have undesirable side effects, leaving an enormous unmet need for efficacious and safe therapy for obesity.

Up to 20 percent of patients with diabetes have a major depressive disorder [26]. Depression in patients with diabetes may be associated with poor compliance with therapy [27], poor glycemic control [28], [29], [30] and an increased risk of complications [31], [32]. As an inhibitor of serotonin re-uptake and one of the world's most prescribed antidepressants, fluoxetin leads to weight loss by decreasing appetite and in turn inhibition of energy intake [33], [34]. However, data on fluoxetine are mainly available among depressed patients and the results are not always consistent [35]. The current meta-analysis is aimed to systematically assess the efficacy of fluoxetine for the treatment of T2DM, concerning the existing studies eligible and available.

## Materials and Methods

Systematical evaluation of the efficacy of fluoxetine to T2DM was based on searching and data-analyzing of the published random, placebo-controlled clinical trials. Body weight loss, fasting plasma glucose, HbA1c, triglyceride and total cholesterol decrement, were compared between fluoxetine and placebo group. Then, the weighted mean difference (*WMD*) and its 95% confidence interval (*CI*) were calculated.

### Study inclusion criteria

1) Studies included should be published, regardless of its research method, publication language or date. 2) Studies included should be random, placebo-controlled trials. 3) The research participants should be Type 2 diabetic patients. 4) *WMD* and its 95% *CI* could be calculated from the raw data extracted from the original literature.

### Search strategy

The literature search, as well as screening of titles, abstracts, and full-text articles, was completed independently by two investigators, according to the inclusion criteria mentioned above. Electronic search was conducted in the database Medline, PubMed, EMBASE, and the Cochrane library, from inception through to March 2011. The terms used for electronic search were fluoxetine OR SSRI OR antidepressants and diabetes OR T2DM OR type 2 diabetes OR obesity OR weight loss OR body weight. Various combinations of the keywords were applied. Moreover, the references of included literature were searched manually and the *Related articles* provided by PubMed were screened.

### Data extraction

Information from each study was extracted independently by two investigators, using a standardized data extraction form. Any dispute was solved unanimously via discussion. The literature approved by both investigators could be included in this meta-analysis. If two or more studies have shared research data, then the study that has the largest amount of samples should be included, while others be excluded. General characteristics of the study (author, year of publication, country, study design, sample size), characteristics of the study groups, their comparability on baseline characteristics (age, sex), duration of treatment, BMI, and outcomes (weight loss, fasting plasma glucose, HbA1c, total cholesterol, triglyceride) were recorded, where available, and double-checked. Where appropriate, an effort was made to complete the data set through communication with the authors.

### Statistical analysis

If *chi-square* test shows there is no significance of heterogenecity among the included studies (*P*>0.10), then fixed model can be applied to calculate *WMD* and its 95% *CI*. By contrast, if there is significant heterogenecity among the included studies (*P*≤0.10), then random model should be applied to calculate *WMD* and its 95% *CI*. Finally, perform *u*-test of *WMD*. The statistics was performed by the software RevMan 4.3.1. The software Stata (version 11.0) was applied for meta-regression analysis.

## Results

### Search results

**Figure 1:**
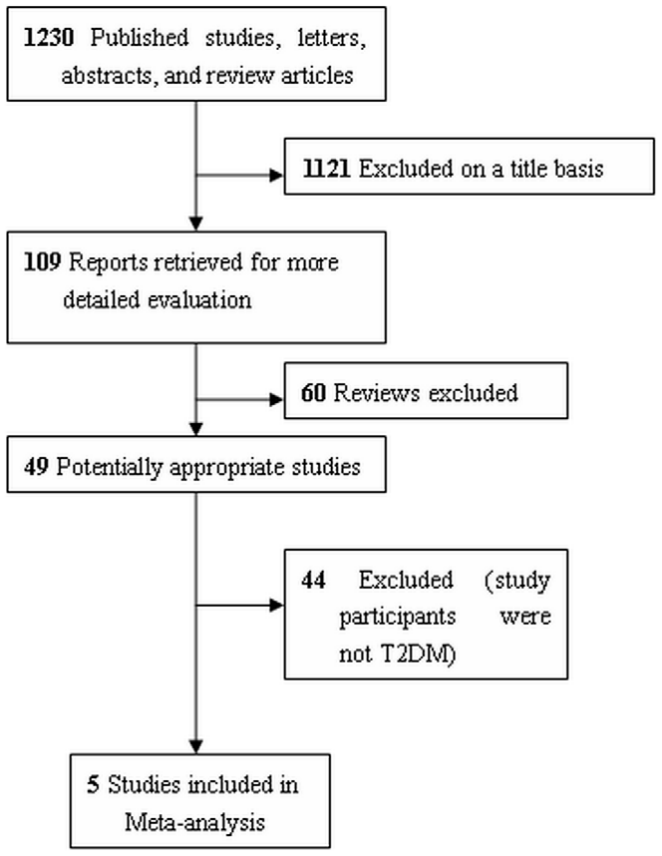
Flow chart of the systematic review and meta-analysis.

The search strategy identified 1230 potentially relevant studies, fifteen of which were searched through reference sections of relevant publications or manual search. A flow chart summarizing search results is provided in Figure 1. One thousand one hundred and twenty-one publications were excluded since it was clear from the title that they did not fulfill the selection criteria. From the remaining 109 publications, 60 reviews were excluded. Forty-nine articles were read in full, independently by two investigators, to assess their accordance with the predefined inclusion criteria. Forty-four studies were excluded because they were performed among depressed or obese participants. Finally, 5 studies were included in the meta-analysis [36], [37], [38], [39], [40], all of which were randomized, placebo-controlled and double-blind parallel clinical trials.

### Systemic review

General characteristics of the eligible studies were given in Table 1. The participants were generally in poor glycemic control [41]. Only one study included insulin-using patients, while the other 4 excluded patients under insulin therapy [40]. The follow-up interval ranged from 6 to 12 months in 4 studies, while the rest one lasted only 2 months [36].

**Table 1:** General characteristics and baseline variables of the included studies.

Study & year	*N*		Country	Follow-up duration (m)	Age(y)		Sex (M/F)		BMI (Kg/m^2^)		HbA1c (%)		FPG (mmol/L)		TG (mmol/L)		TC (mmol/L)	
	F	P			F	P	F	P	F	P	F	P	F	P	F	P	F	P
Gray DS, 1992	24	24	USA	6	54.8±10.9	56.2±8.4	8/16	14/10	38.2±5.7	39.2±7.1	10.54±2.18	10.21±2.96	8.5±3.1	10.8±4.2	U	U	U	U
O'Kane M, 1994	9	10	UK	12	59.6(51–71)	54.9(23–72)	2/7	4/6	36.8(30.7–53.0)	35.8(30.1–43.2)	9.7(7.3–11.8)	9.2(6.1–11.4)	7.95.9–10.2)	7.2(5.6–10.1)	2.15(1.55–3.75)	1.90(1.29–2.28)	6.1(5.8–6.8)	5.4(4.4–6.0)
Breum L, 1995	20	20	Denmark	12	43.6±9.8	44.3±8.7	13/7	15/5	36.9±4.5	39.5±4.7	7.4±2.2	6.8±1.8	10.1±4.4	8.7±2.3	2.7±2.0	2.7±2.2	6.8±0.9	6.5±2.2
Connolly VM, 1995	11	13	UK	6	67	65	10/1	5/8	32.0(28.7–34.9)	31.5(29.8–33.4)	8.0(7.5–9.8)	8.7(7.4–10.7)	7.3(6.3–12.2)	10.2(6.1–11.7)	U	U	U	U
Daubresse JC, 1996	39	43	Belgium	2	52±1	52±2	U	U	34.5±0.7	34.0±0.8	8.5	8.6	U	U	U	U	U	U

F: Fluoxetine group.

P: Placebo group.

U: Unkown.

The previous medication treatment was maintained during the follow-up interval. In O'Kane study, medications for obesity-related problems, such as osteoarthritis of weight bearing joints, hypertension, ischemic heart disease and varicose veins were continued [38]. In Gray study, all subjects had been put on insulin before they were enrolled, and they maintained the insulin therapy [40]. In Connolly study, all participants were diet controlled T2DM. The previous treatment was maintained during the follow-up interval, except medications that could alter weight, such as steroids, appetite suppressants, anti-depressants, diuretics, and thyroid hormone replacement [37]. In Breum study, those who were taking medications that could alter body weight had been excluded from the research. None of the participants received insulin therapy [39]. In Daubresse study, the usual oral hypoglycemic agent therapy was maintained during the rearch [36]. Patients who were concurrently in treatment with other anti-obese drugs had been excluded from all of the 5 studies.

The mean age of participants varied between different studies and ranged from 44 to 67 years. Sex ratio was presented in 4 studies. The dosage of fluoxetine was highly consistent (60 mg daily) among the 5 included studies. At the same time, the controls received placebo in the 5 studies. Two studies involved a dietary intervention for both the fluoxetine and placebo group [37], [38]. None presented information of attrition rate. In the trial by Daubresse, participants were selected only when they had good compliance [36]. No studies discussed allocation concealment (Table 2). Adverse events occurred in both fluoxetine and placebo group. Tremor, sweating and somnolence were reported in very few cases.

**Table 2:** Quality assessment of included studies.

Study	Randomization	Allocation concealment	Blindness	Blindness scheme	ITT analysis	Jadad score
Gray DS	Yes, method unknown	unknown	double-blind	unknown	unknown	2
O'Kane M	Yes, method unknown	unknown	double-blind	unknown	no	2
Breum L	Yes, method unknown	unknown	double-blind	unknown	unknown	2
Connolly VM	Yes, method unknown	unknown	double-blind	unknown	no	2
Daubresse JC	Yes, method unknown	unknown	double-blind	unknown	unknown	2

### Meta-analysis

#### Body weight

**Figure 2:**
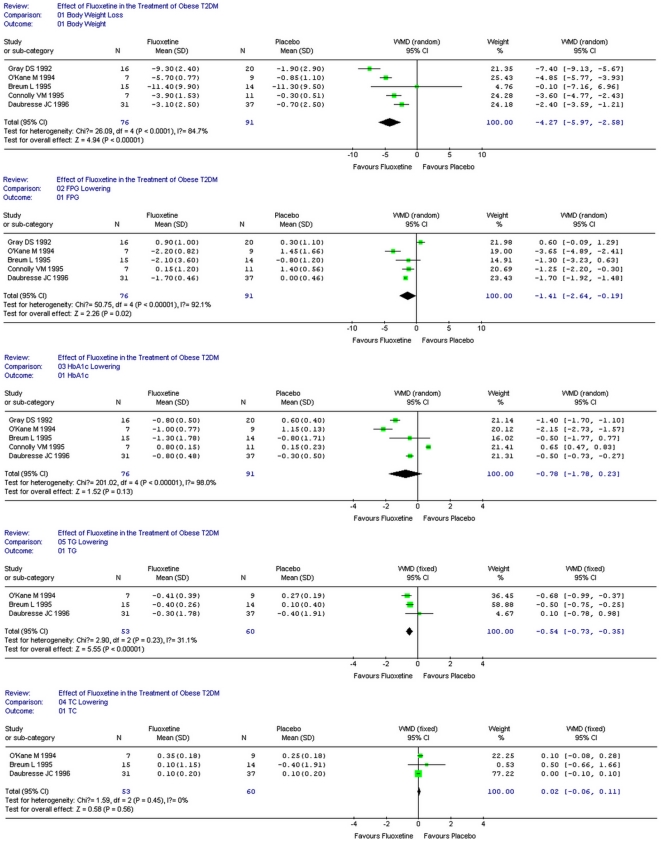
Metabolic effects of fluoxetine in adults with T2DM.

All of the 5 studies reported body weight loss due to fluoxetine. Heterogenecity test of the 5 studies suggested random model be applied (*P*<0.000 1). According to *WMD* calculation (Figure 2), fluoxetine therapy resulted in 4.27 Kg weight loss compared with placebo (95%CI 2.58–5.97, *P*<0.000 01).

#### Fasting plasma glucose

Four studies reported the decrease of fasting plasma glucose (FPG) after fluoxetine therapy, while the rest one claimed the reverse [36]. Heterogenecity test of the 5 studies suggested random model be applied (*P*<0.000 01). According to *WMD* calculation (Figure 2), fluoxetine therapy lead to 1.41 mmol/L of FPG decrement compared with placebo (95%CI 0.19–2.64, *P* = 0.02).

#### HbA1c

Four studies reported the decrease of HbA1c after fluoxetine therapy, while the rest one claimed the reverse [36]. Heterogenecity test of the 5 studies suggested random model be applied (*P*<0.000 01). According to *WMD* calculation (Figure 2), fluoxetine therapy accounted for 0.78% of HbA1c decrement compared with placebo (95%CI−0.23–1.78). However, this effect was not statistically significant (*P* = 0.13).

#### Triglyceride

Two studies reported the decrease of triglyceride (TG) after fluoxetine therapy [36], while one claimed the reverse [36]. Heterogenecity test of the 3 studies suggested fixed model be applied (*P* = 0.23). According to *WMD* calculation (Figure 2), fluoxetine therapy lead to 0.54 mmol/L of TG decrement compared with placebo (95%CI 0.35–0.73, *P*<0.000 01).

#### Total cholesterol

Three studies reported the increase of total cholesterol (TC) after fluoxetine therapy [36]. Heterogenecity test of the 3 studies suggested fixed model be applied (*P* = 0.45). According to *WMD* calculation (Figure 2), fluoxetine therapy lead to 0.02 mmol/L of TC increment compared with placebo (95%CI −0.06–0.11). However, this effect was not statistically significant (*P* = 0.56).

### Meta-regression

Using the net change in the fluoxetine group minus the placebo group, we performed a meta-regression to investigate potential interactions of weight loss, HbA1c, and FPG with study variables including mean age of participants, gender distribution in fluoxetine/placebo group, baseline BMI across studies and fluoxetine/placebo conditions, and duration of treatment. However, none of the combinations approached significance (All *P*>0.05).

## Discussion

Earlier longer term trials with fluoxetine in obese patients showed that the effect of drug on body weight is transient [42], which lead to the discontinuation of the clinical development program of fluoxetine as an anti-obesity drug. In recent years, various adverse effects have been associated with orlistat and sibutramine, two kinds of diet pills which are widely prescribed throughout the world. Therefore, a lot of researchers begin to focus again on fluoxetine [43]. In 2005, a review of pharmacotherapy for weight loss [44] included 6 trials, 4 [36], [37], [38], [40] of which are included in our work. Of the remaining 2 studies, one studied both T2DM and prediabetes [45], while the other one could not be identified through our search strategy. Moreover, the previous review focused on body weight and HbA1c improvement, while our work investigated more clinical variables such as FPG, TC and TG. In another review by Serretti and Mandelli [35], most of the included studies were not placebo-controlled, and comparison with a composite placebo sample derived from other studies was performed. They further demonstrated that estimates from placebo and non-placebo controlled studies were not different, which could probably interpreted by the potential bias linked to the selection of studies. The current meta-analysis has, for the first time, systematically identified and pooled a wide range of evidence about the efficacy of fluoxetine in obese people with T2DM.

In the 5 studies included in current meta-analysis, the participants were not depressed patients complicated with diabetes or obesity, but T2DM patients from diabetic clinic. Our analysis demonstrated that fluoxetine produced significant and clinically meaningful changes in body weight, FPG, HbA1c, triglyceride and cholesterol in T2DM, which confirmed that the metabolic benefit of fluoxetine was independent of its anti-depressive effect. Body weight loss has positive effects on metabolic control in obese patients with T2DM. Moreover, the metabolic benefits acquired from pharmacotherapy can be sustainable after steady weight loss. In the 5 included studies, few severe adverse events were reported. Fluoxetine were generally well tolerated and produced a low incidence of serious adverse events. By contrast, the use of orlistat has been associated with lower levels of fat soluble vitamins and supplementation, while sibutramine produced palpitations and a nonsignificant increase in pulse rate [42]. Fluoxetine is effective and safer in treating obesity with T2DM, compared with other pharmacologic therapies.

In current meta-analysis, the treatment duration was up to 12 months. Therefore, the long-term effect of fluoxetine in obese patients with T2DM is still unclear. However, a retrospective cohort study has shown that the benefits of weight loss in T2DM may be long-lasting [46]. Though considering psychiatric patients, Serretti and Mandelli [42], showed a trend for an increase of weight with fluoxetine in the medium-long term treatment. Among the 5 studies, almost all of the participants are middle-aged, who are generally in poor glycemic control. Since BMI was reported, it was accurate to assess the degree of overweight. As the participants might be intentionally selected, and some studies eliminated the noncompliant participants, our analysis may have selection bias, and should be considered appropriate only to similar populations.

Fluoxetine exhibited modest and significant weight loss effect. Even moderate weight loss can lead to considerable blood pressure and glucose improvement in obese patients with T2DM. In non-diabetic populations, the efficacy of weight-loss drugs on body weight is also modest, and the weight is easily regained after the medication is discontinuated [47]. This phenomenon is more obvious in diabetic patients, which may explain the small reduction in weight noted in our analysis.

Fluoxetine showed statistically significant effects on FPG, which is very meaningful because FPG is an important indicator that can be related to the development of the disease. The trend towards greater improvement in glycemic control with fluoxetine could not be attributed simply to the effects of weight loss. It is possible that fluoxetine affects other unmeasured characteristics, such as insulin action.

Fluoxetine therapy led to significant reduction in triglyceride levels in three studies [36], [38], [39]. The improvement of lipid profiles is encouraging, given that they are associated with risk of ischemic heart disease [48]. However, it remains unclear whether the improved lipid profiles could be sustained in long-term period.

Concealing allocation was not mentioned in any study, nor did the method of randomization. The method of sampling and participant recruitment was rarely described, making it difficult to conclude to whom the intervention can be applied.

In general practice, weight loss medications are usually combined with various lifestyle modification [49]. However, no study in current analysis examined the efficacy of pharmacotherapy combined with lifestyle or behavioral interventions. Some studies reported that addition of one kind of behavioral intervention may improve the effectiveness of medication in the obese [50].

It is noteworthy that T2DM is a progressive disease. If obesity is not intervened, the disease will become more irreversible with time. Therefore, various potential approaches of weight loss need to be evaluated. While lifestyle modification alone is not enough, weight loss pharmacotherapy should be applied in conjunction with behavioral programs. Although in two trials of fluoxetine, the follow-up duration lasted for almost one year [38], [39], longer term of follow-up and larger study population are needed.

Further work is needed to examine the combination of lifestyle modification and pharmacotherapy in order to determine the optimal dosage and sequence of the two kinds of intervention. More concern should be attached on the progress of fluoxetine in the treatment of obese with T2DM. The research is expected to lead to the development of an effective, safe and long-term pharmacotherapy for obese patients with T2DM.
